# Korean American Church Leaders as Mental Health Gatekeepers in the USA: A Needs Assessment of Readiness, Barriers, and Referrals

**DOI:** 10.1007/s10943-026-02589-3

**Published:** 2026-02-22

**Authors:** Nari Yoo, Michelle Yujin Kang, Erin Joohee Kim, Soe Young Lee, Jiwon Woo, Samuel Yi Kim

**Affiliations:** 1 School of Social Work, University of Michigan, 1 1080 S University Ave, Ann Arbor, MI 48109 USA; 2 Social-Personality Psychology Program, University of Rochester, 301 Meliora Hall, Rochester, NY 14627 USA; 3 Lynch School of Education and Human Development, Boston College, 140 Commonwealth Ave, Chestnut Hill, MA 02467 USA; 4 Wheelock College of Education and Human Development, Boston University, 621 Commonwealth Ave, 207A, Boston, MA 02215 USA; 5 Mustard Seed Generation, 539 W Commerce St Ste 3036, Dallas, TX 75208 USA; 6 School Psychology Specialist Program, Texas Woman’s University, 1315 N. Bell Ave. CFO 702, Denton, TX 76204 USA

**Keywords:** Korean Americans, Mental health, Church leaders, Help-seeking, Stigma, Gatekeepers

## Abstract

Asian Americans underutilize mental health services, with particularly low rates among Korean American (KA) immigrants. Churches serve as central sources of support in this community, positioning clergy as critical mental health gatekeepers. Yet little is known about the factors shaping KA church leaders’ readiness, perceptions, and referral behaviors. This study analyzed survey data from 93 KA church leaders across the USA to explore factors associated with readiness (comfort addressing mental health), perceptions of issues and barriers, and the action of recommending professional counseling. Using multiple regressions, we found that prior mental health training was the only significant predictor of leader readiness, with trained leaders nearly three times more likely to report higher comfort levels (OR = 2.97, *p* < 0.05). Perceptions of church needs varied by ministry context and personal experience: Leaders with mental health training were over six times more likely to recognize depression and suicide as key concerns (OR = 6.71, *p* < 0.01) and more than twice as likely to identify marriage conflict (OR = 3.11, *p* < 0.05). In contrast, leaders who had personally received counseling were significantly less likely to report depression and suicide as a congregational issue (OR = 0.23, *p* < 0.05). In the integrative model, referral behavior was shaped primarily by ministry context and stigma awareness. Leaders in Korean-speaking ministries were significantly less likely to recommend counseling (OR = 0.02, *p* < 0.05), while those who identified stigma as a major issue were substantially more likely to make referrals (OR = 9.50, *p* < 0.05). These findings highlight the central role of training, bicultural ministry contexts, and stigma recognition in shaping church leaders’ engagement with mental health. The study underscores the need for culturally adapted training programs and closer collaboration between mental health professionals and immigrant churches to address persistent disparities in KA communities.

## Introduction

The National Latino and Asian American Study found that only 8.6% of Asian Americans sought mental health-related services, with 34.1% of those with probable diagnoses accessing any services (Abe-Kim et al., 2007). This underutilization is especially pronounced among immigrants, with US-born Asian Americans showing higher service use rates than their immigrant counterparts (Abe-Kim et al., 2007; Le Meyer et al., 2009). Among Korean American (KA) immigrants specifically, only 8.5% reported utilizing mental health services despite 23% experiencing depressive symptoms (Park et al., 2013). Cultural beliefs and lack of culturally competent services contribute to low recognition of mental health problems and service utilization, while key barriers include language preferences, confidentiality concerns, and affordability (Koh et al., 2021). Immigration-related factors, including shorter US residence duration and English proficiency, significantly influence service utilization patterns (Le Meyer et al., 2009; Park et al., 2013).

These barriers suggest that KAs require approaches that better address their needs. One approach is to collaborate with trusted leaders in the KA community, such as church leaders, to overcome these barriers and facilitate access to mental health support. Given that more than two-thirds of KAs identify as Christians, Korean clergy play influential roles in their daily lives and represent a crucial resource for addressing mental health disparities (Cheon et al., 2016; Lee et al., 2008). KA churches have traditionally served as a primary source of social and emotional support in this community (Baek et al., 2024), and some pastors have even reported spending 30–50% of their ministerial time addressing the health and social needs of their members (Jo et al., 2010). Clergy also reported feeling responsible for their congregants’ well-being, while demonstrating varying levels of mental health literacy (Jang et al., 2017).

In this context, KA clergy serve as crucial gatekeepers for mental health services in their communities, yet face significant challenges in addressing congregants’ mental health needs. KAs are more likely to seek guidance from church leaders than traditional mental health services since talking about mental health is considered “taboo,” making clergy perspectives critical for community mental health (Han et al., 2023). However, research reveals that KA pastors experience barriers, including difficulty assessing mental health issues, limited knowledge of available mental health counseling services for referrals, and challenges integrating mental health concerns with religious beliefs (Cheon et al., 2016). For example, because clergy are seen as moral or spiritual authorities, admitting their own mental health struggles may threaten their credibility, trust, or “face” (*chaemyeon*, 체면) within the community, that is, their social dignity and moral standing rooted in the cultural expectation to maintain respectability and avoid shame (Baek et al., 2024).

These barriers are compounded by broader systemic issues affecting the KA community, including language barriers, cultural differences, financial constraints, limited transportation, and perceived stigma (Wu et al., 2009). Faith-based organizations are also reported to depend on a range of technical assistance to ensure the provision of essential health services, particularly in under-resourced neighborhoods (Kearns, 2006). Non-profit organizations, such as Mustard Seed Generation (MSG), have collaborated with local KA communities and churches to offer a range of support. In this spirit, MSG created and administered a needs assessment to understand the areas of concern for KA communities and to determine the best way to equip these church leaders.

We draw on two complementary models to examine church leaders’ mental health gatekeeping. First, the mental health literacy literature (Jorm, 2012; Kutcher et al., 2016) encompasses the knowledge and beliefs about mental health that enable recognition of disorders, help-seeking, and support for others. Mental health literacy includes the ability to recognize mental health problems, knowledge of available treatments and resources, and attitudes that promote help-seeking. Second, the community gatekeeper model (Isaac et al., 2009; Wyman et al., 2008) describes how community leaders who have regular contact with at-risk individuals can identify warning signs and facilitate access to care. This model emphasizes gatekeepers’ willingness to engage, capacity to recognize problems, and ability to take referral actions.

### The Present Study

**Fig. 1:**
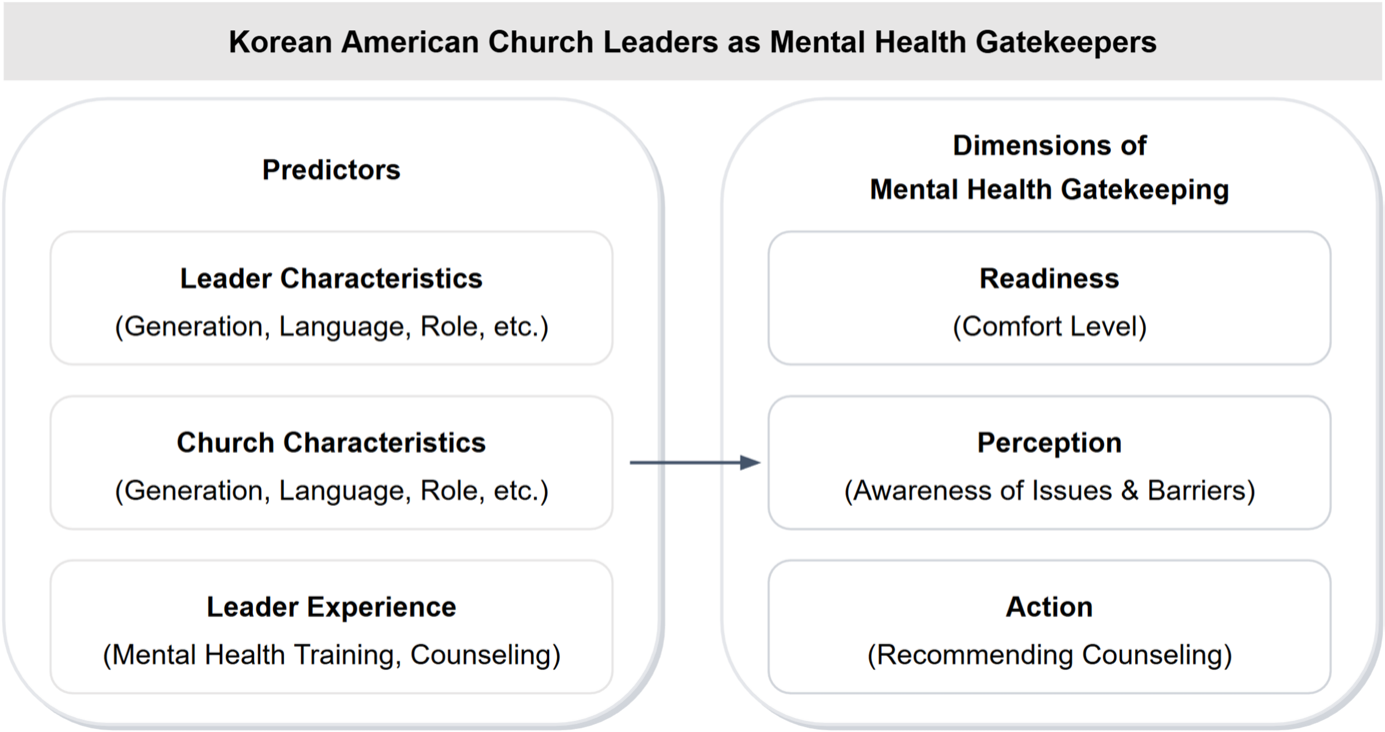
Dimensions of mental health gatekeeping among Korean American church leaders

The present study utilizes a national needs assessment of KA church leaders to address these gaps. As an exploratory study, we examined three dimensions of mental health gatekeeping among Korean American church leaders: (1) readiness to refer congregants to mental health services, reflecting willingness and openness to facilitate help-seeking; (2) perception of mental health problems among congregants, reflecting recognition capacity; and (3) action taken to promote mental health awareness, reflecting concrete helping behaviors. While these dimensions may interact and inform one another, we examined them as distinct outcomes to identify their unique predictors and practical implications for intervention development (Fig. 1).

## Methods

### Data and Participants

This study utilized a cross-sectional quantitative design to analyze data from a national online needs assessment of KA church leaders. The survey was administered between November 2021 and January 2022. Participants were recruited through a combination of convenience and snowball sampling strategies, targeting individual church leaders, denominational listservs, and other community networks across the USA. The inclusion criteria required participants to be at least 18 years old and to have served in a leadership role within a KA church for at least 1 year. The final analytic sample for this study consisted of 93 leaders. The survey instrument was an 18-item questionnaire developed via several rounds of Research Committee meetings within the community organization, Mustard Seed Generation, to capture a range of information related to church leadership and mental health. This needs assessment tool was designed to evaluate self-reported comfort, perceptions, and practices rather than to validate psychometric constructs. All variables for this study were derived from participant self-reports. The survey was offered in both English and Korean. The full survey instrument is attached to Appendix A.

### Measures

#### Dependent Variables

##### Readiness (Comfort Level)

To measure a leader’s psychological readiness, participants responded to the question, “How comfortable are you with addressing mental health issues in your church?” Responses were captured on a 4-point Likert-type scale, with options including uncomfortable, neutral, comfortable, and very comfortable. The higher scores indicate higher readiness. This variable was treated as an ordered categorical outcome.

##### Perception (Awareness of Issues and Barriers) 

To assess how leaders perceive the specific needs and challenges within their communities, they were presented with two checklists. The first asked, “List all the issues that you are concerned about in your church,” and provided a list of potential issues (e.g., depression and suicidal ideation, marriage conflict, racism, mental health stigma). The second question asked, “Select all the stumbling blocks you experience when handling the issues,” and provided a list of potential barriers (e.g., “I don’t know how”, “People don’t share”). For the perception outcome, each item was operationalized as a separate binary variable (1 = selected, 0 = not selected).

##### Action (Recommending Counseling)

To measure a key gatekeeping behavior, we used a binary variable derived from the question assessing whether a leader had ever recommended mental health counseling services to a congregant (1 = yes, 0 = no).

#### Independent Variables

##### Leader Characteristics

This category included demographic and functional role variables. (1) Immigration generation was coded as a categorical variable with three groups: 1st generation (reference), 1.5 generation (individuals born abroad who immigrated during childhood or adolescence; Rumbaut, 2004), and 2nd/3rd generation combined. (2) Years in current role was measured as a continuous variable reflecting the length of tenure in the respondent’s present leadership position. (3) Leadership positions were originally measured through a multiple-choice survey item in which respondents selected all roles they held in their church, including pastors, assistant or youth pastors, elders, deacons, small-group leaders, Sunday school teachers, and other ministry roles such as council chairpersons, preschool or education directors, or staff spouses. These individual positions were then aggregated into three binary indicators: pastoral staff (senior, assistant, youth pastors, and ministry directors); governing lay leaders (elders, deacons, and council chairpersons); and teaching/fellowship leaders (small-group leaders, Sunday school teachers, and comparable fellowship or ministry leaders).

##### Church Characteristics

This category included variables describing the organizational context. Denomination was a categorical variable (Presbyterian/reformed [reference group], Baptist, Methodist, and Nondenominational/independent). Congregation size was measured ordinally (< 50 [reference group], 50–100, 100–200, 200–500, and 500 +). The ministry language context was captured using two separate binary variables: primary involvement in the Korean-speaking Ministry (KM) and in the English-speaking Ministry (EM). Two additional binary variables captured involvement in specific ministry areas: Ministry serving children/youth and Missions Ministry.^1^

##### Leader Experience

Two binary variables measured prior mental health-related experiences: having received any form of mental health training and having personally received professional counseling.

### Analytic Strategy

Followed by descriptive statistics, we executed a regression analysis to address our core research questions. First, to assess leader readiness, an ordered logistic regression was used to model the factors associated with a leader’s self-reported comfort level. Second, to investigate perception, a series of binary logistic regressions was conducted to determine how the full set of leader, church, and experience variables predicted the identification of each specific church issue and ministry barrier. Finally, to understand action, an integrative binary logistic regression was constructed to identify the factors associated with recommending mental health counseling. This final model included all leader, church, and experience variables, as well as the leader’s comfort level and their perceptions of the selected issues and barriers, to identify the ultimate drivers of this key support behavior. All regression results are reported as odds ratios (ORs) with 95% confidence intervals. All analyses were conducted using Stata/MP 17.0. Statistical analyses were conducted by a researcher with formal training in quantitative methodology (first author).

## Results

### Descriptive Characteristics of the Sample

The demographic, ministry, and mental health experience characteristics of the 93 KA church leaders are presented in Table 1. The sample was predominantly first-generation (55.9%), with Korean as the preferred language (49.5%). The most common denominational affiliation was Presbyterian/Reformed (47.3%). Leaders served in a variety of functional roles, with a majority identifying as pastoral staff (71.0%). In terms of church context, 49.5% of leaders served in primarily Korean-speaking ministries (KM focused), while 36.6% served in bilingual contexts. A majority of participants (52.7%) reported having received some form of mental health training, while a minority (38.7%) had ever personally received mental health counseling.

**Table 1:** Descriptive characteristics of the sample (*N* = 93)

Characteristic	Category	Frequency (N)	Percentage (%)
*Leader characteristics*			
Immigration generation	1st Generation	52	55.91
	1.5 Generation	23	24.73
	2nd/3rd Generation	18	19.35
Preferred language	English	26	27.96
	Korean	46	49.46
	Fluent in both (bilingual)	21	22.58
Role: pastoral staff	Yes	66	70.97
Role: governing lay leader	Yes	44	47.31
Role: relational/teaching leader	Yes	45	48.39
Years in current role	Mean (Std. Dev.)	9.99 (8.53)	–
*Church characteristics*			
Denomination	Presbyterian/reformed	44	47.31
	Baptist	25	26.88
	Methodist	9	9.68
	Nondenominational/independent	15	16.13
Congregation size	Less than 50	26	27.96
	50–100	15	16.13
	100–200	18	19.35
	200–500	21	22.58
	500 +	13	13.98
Primary ministry focus	KM only	46	49.46
	EM only	13	13.98
	Bilingual/both	34	36.56
Ministry: child/youth	Yes	53	56.99
Ministry: missions	Yes	26	27.96
*Leader mental health experience*			
Received mental health training	Yes	49	52.69
Ever received professional counseling	Yes	36	38.71

### Factors Associated with Leader Readiness (Comfort Level)

Our first research question sought to identify the factors associated with a leader’s psychological readiness, defined as their self-reported comfort level with mental health topics. Table 2 presents the results of the ordered logistic regression model. After controlling for a set of leader, church, and experience variables, the analysis revealed a single, powerful predictor: prior mental health training. Leaders who had received training had nearly 3 times the odds of reporting higher comfort levels than those without training (OR = 2.97, *p* = 0.019). No other characteristics, including a leader’s generational status, years in ministry, role, or specific church context, were significantly associated with their readiness.

**Table 2:** Full ordered logistic regression model predicting leader readiness (comfort level) (*N* = 93)

Predictor	Odds ratio (OR)	Std. Err	z	P >|z|	[95% Conf. Interval]
**Received MH training (Yes)**	**2.971**	**1.378**	**2.35**	**0.019**	**[1.197, 7.373]**
Generation: 1.5 Gen	0.552	0.351	−0.93	0.350	[0.158, 1.923]
Generation: 2nd/3rd Gen	1.120	0.870	0.15	0.884	[0.244, 5.135]
Pastoral staff	1.810	1.079	1.00	0.319	[0.563, 5.820]
Governing lay leader	1.372	0.728	0.60	0.551	[0.485, 3.883]
Teaching/fellowship leader	1.065	0.626	0.11	0.915	[0.337, 3.369]
Years in role	1.033	0.029	1.14	0.254	[0.977, 1.092]
Denomination: Baptist	1.111	0.665	0.18	0.861	[0.344, 3.589]
Denomination: Methodist	1.015	0.798	0.02	0.985	[0.217, 4.744]
Denomination: Nondenominational	1.462	1.055	0.53	0.599	[0.355, 6.017]
Congregation size: 50–100	1.444	1.044	0.51	0.611	[0.350, 5.956]
Congregation size: 100–200	0.591	0.395	−0.79	0.432	[0.159, 2.192]
Congregation size: 200–500	0.973	0.725	−0.04	0.971	[0.226, 4.194]
Congregation size: 500 +	0.462	0.359	−0.99	0.320	[0.101, 2.120]
Ministry: KM	0.541	0.359	−0.93	0.354	[0.147, 1.987]
Ministry: EM	0.711	0.500	−0.49	0.627	[0.179, 2.824]
Ministry: child/youth	1.081	0.721	0.12	0.907	[0.292, 3.998]
Ministry: missions	1.352	0.760	0.54	0.592	[0.449, 4.068]
Received counseling (Yes)	1.113	0.539	0.22	0.825	[0.431, 2.877]

### Leader Profiles and the Perception of Church Needs

We further examined how the full range of leader and church characteristics shapes perceptions of specific needs and barriers. The results are summarized in Table 3 (issues) and Table 4 (barriers). The perception of church issues was significantly shaped by a complex interplay of ministry context, generational status, and personal experience (Table 3). Concern for racism was powerfully predicted by ministry environment; leaders in English Ministries had over 33 times the odds (OR = 33.29, *p* < 0.01), and those in Missions Ministry had over 10 times the odds (OR = 10.53, *p* < 0.01) of identifying racism as a concern. This was also strongly associated with serving mid- to large-sized congregations (100–500 members). Perceptions of mental health stigma were also significantly higher among leaders involved in Missions Ministry (OR = 5.26, *p* < 0.05) and those serving in Korean-speaking Ministries (OR = 4.56, *p* < 0.05). A leader’s personal experience was a critical predictor of clinical issues. Mental health training was the sole significant predictor for being concerned about marriage conflict (OR = 3.11, *p* < 0.05) and was also strongly associated with a heightened concern for depression and suicide (OR = 6.71, *p* < 0.01). Conversely, leaders who had personally received mental health counseling were significantly less likely to report depression and suicide as a church-wide problem (OR = 0.23, *p* < 0.05).

**Table 3:** Full logistic regression models predicting perception of church issues (*N* = 93)

Predictor	Racism	Marriage conflict	Depression/suicide	Mental health stigma
	OR (SE)	OR (SE)	OR (SE)	OR (SE)
*Leader characteristics*				
Generation: 1.5 Gen	0.27 (0.27)	0.29 (0.24)	1.11 (0.92)	1.63 (1.41)
Generation: 2nd/3rd Gen	7.74 (9.41)	0.73 (0.74)	4.35 (5.10)	17.31 (20.82)*
Pastoral staff	0.41 (0.37)	2.07 (1.54)	1.34 (1.01)	2.11 (1.73)
Governing lay leader	0.27 (0.23)	0.99 (0.66)	0.48 (0.34)	0.21 (0.17)*
Teaching/fellowship leader	1.45 (1.31)	0.66 (0.51)	2.56 (2.11)	2.12 (1.97)
Years in role	0.99 (0.04)	1.03 (0.04)	1.07 (0.04)*	0.97 (0.04)
*Church characteristics*				
Denomination: Baptist	0.22 (0.24)	0.38 (0.30)	0.59 (0.46)	0.94 (0.73)
Denomination: Methodist	1.57 (1.63)	0.29 (0.27)	0.99 (0.95)	3.17 (3.06)
Denomination: Nondenominational	0.19 (0.20)	1.09 (1.00)	0.43 (0.45)	0.43 (0.41)
Congregation size: 50–100	0.19 (0.22)	0.31 (0.30)	0.94 (0.96)	0.33 (0.36)
Congregation size: 100–200	8.97 (9.81)*	0.84 (0.76)	1.72 (1.56)	1.43 (1.29)
Congregation size: 200–500	20.78 (28.01)*	0.98 (0.97)	5.49 (5.71)	0.91 (0.96)
Congregation size: 500 +	1.57 (2.02)	1.49 (1.49)	3.48 (3.47)	0.99 (0.97)
Ministry: KM	0.16 (0.16)*	0.28 (0.24)	0.81 (0.68)	4.56 (4.13)*
Ministry: EM	33.29 (42.27)**	3.73 (3.45)	2.05 (1.84)	0.79 (0.73)
Child/youth ministry	0.08 (0.09)*	1.09 (0.92)	2.84 (2.34)	1.45 (1.28)
Missions Ministry	10.53 (9.52)**	0.64 (0.46)	0.59 (0.48)	5.26 (4.16)*
*Leader experience*				
MH training (Yes)	0.32 (0.23)	3.11 (1.79)*	6.71 (4.24)**	1.73 (1.03)
Counseling (Yes)	0.29 (0.22)	0.42 (0.26)	0.23 (0.16)*	0.49 (0.35)

Results are presented as Odds Ratios (standard errors). Reference groups for categorical variables are 1st Gen, Presbyterian/reformed, < 50 members, and KM focused (for individual ministry variables). *p < 0.05, **p < 0.01, ***p < 0.001

**Table 4:** Full logistic regression models predicting perceived ministry barriers (*N* = 93)

Predictor	“I Don’t Know How”	“People Don’t Share”
	OR (SE)	OR (SE)
*Leader characteristics*		
Generation: 1.5 Gen	2.73 (2.51)	0.30 (0.26)
Generation: 2nd/3rd Gen	2.48 (2.81)	1.11 (1.00)
Pastoral staff	0.34 (0.30)	1.64 (1.22)
Governing lay leader	3.27 (2.72)	1.14 (0.74)
Teaching/fellowship leader	2.63 (2.25)	0.78 (0.59)
Years in role	0.85 (0.06)*	1.00 (0.03)
*Church characteristics*		
Denomination: Baptist	11.44 (12.39)*	1.64 (1.19)
Denomination: Methodist	0.37 (0.37)	0.41 (0.40)
Denomination: Nondenominational	0.30 (0.36)	1.36 (1.15)
Congregation size: 50–100	46.24 (59.53)**	1.15 (0.97)
Congregation size: 100–200	17.01 (20.90)*	0.84 (0.66)
Congregation size: 200–500	1.50 (1.68)	2.90 (2.72)
Congregation size: 500 +	0.29 (0.36)	0.14 (0.14)
Ministry: KM	1.20 (1.13)	2.74 (2.22)
Ministry: EM	0.05 (0.05)**	0.38 (0.32)
Child/youth ministry	7.67 (7.95)*	1.00 (0.78)
Missions Ministry	0.95 (0.88)	2.50 (1.68)
*Leader experience*		
MH training (yes)	5.95 (4.66)*	1.26 (0.70)
Counseling (yes)	0.79 (0.57)	1.40 (0.81)

Results are presented as Odds Ratios (Standard Errors). Reference groups for categorical variables are 1st Gen, Presbyterian/reformed, < 50 members, and KM focused (for individual ministry variables). *p < 0.05, **p < 0.01, ***p < 0.001

The analysis of ministry barriers revealed distinct patterns (Table 4). The internal barrier of “I don’t know how” was significantly predicted by both experience and context. Each additional year in a role decreased the odds of feeling unequipped (OR = 0.85, *p* < 0.05). Leaders in EM contexts felt significantly more prepared (OR = 0.05, *p* < 0.01), while those in Child/Youth ministries (OR = 7.67, *p* < 0.05) and Baptist denominations (OR = 11.44, *p* < 0.05) were more likely to report this barrier. Mental health training was again a significant factor, increasing a leader’s odds of feeling equipped by nearly 6 times (OR = 5.95, *p* < 0.05). In contrast, the model predicting the external barrier of “People don’t share” yielded no statistically significant predictors, suggesting it is a universally perceived challenge.

### Factors Associated with Recommending Professional Counseling

The final integrative logistic regression model sought to identify the factors associated with recommending mental health counseling, controlling for all other variables (Table 5). The analysis revealed that the decision to refer is most significantly predicted by a leader’s specific ministry context and their awareness of systemic barriers. Leaders serving in Missions Ministry had over 32 times the odds of recommending mental health counseling compared to those not involved in missions (OR = 32.22, *p* = 0.035). Conversely, leaders in primarily Korean-speaking ministries had significantly lower odds of making a referral (OR = 0.02, *p* = 0.027). Leaders who identified mental health stigma as an issue had 9.5 times the odds of recommending mental health counseling (OR = 9.50, *p* = 0.027). Leaders from Methodist denominations were significantly less likely to make referrals compared to their Presbyterian/reformed counterparts (OR = 0.02, *p* = 0.048). Leaders’ reported readiness (comfort level) and perceptions of issues were not significant factors associated with recommending mental health counseling.

**Table 5:** Full final logistic regression model predicting action of recommending counseling (N = 93)

Predictor	Coeff. (β)	Std. Err	z	P >|z|	Odds ratio (OR)
*Leader characteristics*					
Generation: 1.5 Gen	0.301	1.115	0.27	0.788	1.35
Generation: 2nd/3rd Gen	1.620	1.628	1.00	0.319	5.05
Pastoral staff	−0.061	1.123	−0.05	0.957	0.94
Governing lay leader	−0.324	0.899	−0.36	0.718	0.72
Teaching/fellowship leader	2.012	1.171	1.72	0.086	7.48
Years in role	0.061	0.063	0.98	0.328	1.06
*Leader mental health experience*					
MH training (yes)	1.127	0.881	1.28	0.201	3.09
Mental health counseling (yes)	1.060	1.039	1.02	0.308	2.89
*Church characteristics*					
Denomination (ref: Presbyterian/reformed)					
Denomination: Baptist	1.610	1.373	1.17	0.241	5.00
Denomination: Methodist	**-3.698**	**1.871**	**-1.98**	**0.048**	**0.02**
Denomination: Nondenominational	−1.699	1.291	−1.32	0.188	0.18
Congregation size (ref: less than 50)					
Congregation size: 50–100	2.204	1.725	1.28	0.201	9.06
Congregation size: 100–200	2.198	1.790	1.23	0.219	9.01
Congregation size: 200–500	0.212	1.605	0.13	0.895	1.24
Congregation size: 500 +	−0.107	1.443	−0.07	0.941	0.90
Ministry: KM	**-3.940**	**1.784**	**-2.21**	**0.027**	**0.02**
Ministry: EM	−1.423	1.244	−1.14	0.252	0.24
child/youth ministry	−2.090	1.202	−1.74	0.082	0.12
Ministry: missions	**3.473**	**1.648**	**2.11**	**0.035**	**32.22**
*Readiness and perception factors*					
Comfort level	0.428	0.519	0.83	0.409	1.54
Issue: racism	−1.076	1.184	−0.91	0.364	0.34
Issue: marriage conflict	1.600	0.910	1.76	0.079	4.95
Issue: depression/suicide	0.698	0.908	0.77	0.442	2.01
Issue: mental health stigma	**2.251**	**1.019**	**2.21**	**0.027**	**9.50**
Barrier: don’t know how	−0.114	0.873	−0.13	0.896	0.89
Barrier: people don’t share	0.085	0.901	0.09	0.925	1.09

Results are presented as Coefficients (β) and Odds Ratios (OR). Reference groups for categorical variables are 1st Gen, Presbyterian/reformed, < 50 members, and KM focused (for individual ministry variables). *p < 0.05, **p < 0.01, ***p < 0.001

## Discussion

### Primary Findings

This study aimed to identify the factors that contribute to KA church leaders’ readiness, perceptions, and actions regarding mental health needs within their congregations. The first major finding is the significant impact of targeted training on a leader’s psychological readiness. Our initial model revealed that prior mental health training was the sole significant predictor of a leader’s comfort level. This result suggests that readiness is not a fixed trait inherent to a leader’s role or experience but is a malleable attribute that can be directly cultivated. Research consistently demonstrates that mental health training significantly enhances the preparedness and effectiveness of clergy as informal mental health gatekeepers. Pickard (2012) found that clergy with more mental health training, better relationships with mental health professionals, and greater knowledge of resources felt significantly more prepared for counseling responsibilities. Similarly, Vermaas et al. (2017) identified that higher numbers of clinical mental health training courses were significant factors associated with improved mental health literacy among clergy, alongside female gender. The impact of training extends beyond individual preparedness to behavioral change and resource utilization. Yamada et al. (2012) also found that factors positively influencing clergy’s mental health referral behavior include prior mental health education, knowledge of mental illness, higher levels of education, and time spent providing individual counseling. This finding highlights that mental health training is a prerequisite for effective gatekeeping among KA church leaders.

Our finding that prior mental health training strongly predicts gatekeeping aligns with broader evidence supporting gatekeeper training programs. Mental Health First Aid (MHFA), designed to help community members identify and respond to mental health crises, has demonstrated effectiveness in increasing knowledge, reducing stigma, and improving confidence in providing support across diverse settings (Jorm et al., 2010; Kitchener & Jorm, 2006; Morgan et al., 2018). Similarly, suicide prevention programs such as Question, Persuade, Refer (QPR) have shown positive outcomes when implemented with faith leaders and community gatekeepers (Burnette et al., 2015; Matthieu et al., 2008). However, existing training programs often lack cultural and religious adaptation. Faith-based mental health trainings that incorporate theological frameworks and address stigma within religious contexts have shown promise (Payne, 2014; Stanford & Philpott, 2011), yet few are tailored to immigrant communities or non-English-speaking populations. For Korean American churches specifically, culturally responsive training should address unique contextual factors including language barriers, collectivist values, hierarchical church structures, and culturally specific expressions of distress (Jang et al., 2017; Kim & Kim, 2025; Lee et al., 2014). Future research should develop and evaluate church-based training models that integrate cultural humility, religious literacy, and community-specific mental health needs to maximize engagement and sustainability among Korean American church leaders.

The second set of findings revealed that both ministry context and personal experience shape a leader’s perception of specific needs. The analysis showed that leaders in cross-cultural or externally focused settings (i.e., Missions Ministry) exhibited a heightened awareness of systemic issues. For instance, involvement in Missions Ministry was a significant predictor of concern about racism and mental health stigma, suggesting that leaders operating at the boundaries of their immediate cultural context may be more attuned to societal forces affecting congregant well-being. Furthermore, a leader’s personal history was a key factor in their success. Leaders with mental health training were significantly more likely to identify depression and suicide as a concern, indicating that training may provide the framework to recognize clinical-level distress. Conversely, leaders who had personally received mental health counseling were significantly less likely to identify depression as a church-wide problem. This may suggest that personal therapeutic experience fosters a more nuanced assessment, enabling leaders to differentiate between individual struggles and broader congregational crises, thereby providing effective pastoral care. For example, Landes (2010) emphasizes the importance of exercising discernment in pastoral care during crises, especially when individuals utilize narratives to process their experiences. Layzell (2019) further notes that, in the context of collective tragedies, pastoral leaders face unique challenges that cannot be addressed solely through individual discernment. In such circumstances, supervision and structured support systems are crucial to safeguard pastoral caregivers and prevent secondary traumatic stress.

Thirdly, denominational differences in our data were limited but notable, with Methodist leaders significantly less likely to recommend mental health counseling than Presbyterian/reformed counterparts, and Baptist leaders disproportionately reporting “I don’t know how” as a barrier. While prior work suggests that denominational identity per se is not a strong predictor of clergy mental health literacy across broad US samples (Hodge et al., 2020), denominational governance structures and institutional cultures may shape how Korean American (KA) churches engage with external resources. Presbyterian/reformed congregations, which comprise nearly half of KA churches nationally (Liu, 2012), are often embedded in larger denominational networks that can provide referral linkages and training opportunities. In contrast, the United Methodist Church (and many Methodist bodies) operates under a connectional polity, organized into local congregations, districts, annual conferences, and general/jurisdictional bodies, meaning that authority, accountability, and pastoral appointments move through these tiers (United Methodist Church, 2016). It may inadvertently reinforce internal care pathways and reduce external referral. Baptist churches, characterized by congregational autonomy (Grenz, 2002), often lack standardized referral systems, leaving leaders uncertain about how to connect congregants with professional care. We suggest that denominational effects in KA churches are best understood as proxies for organizational infrastructure, training pipelines, and the permeability of church–community boundaries, rather than as fixed theological positions.

The final finding emerged from our model, which predicts the action of referring to a congregant to mental health counseling. The analysis revealed that this action was significantly predicted by a specific combination of ministry context and a leader’s awareness of cultural barriers. Specifically, leaders serving in Missions Ministry were significantly more likely to make referrals, perhaps because they encounter complex issues of trauma and crisis that more clearly exceed the bounds of traditional pastoral care. This was coupled with the finding that leaders who perceived mental health stigma as a key issue were also significantly more likely to recommend mental health counseling. This suggests a critical decision-making process: A referral is more likely to occur when a leader not only recognizes a need but also understands that a primary cultural barrier (i.e., stigma) may prevent that need from being effectively met within the church’s internal systems. This finding aligns with extensive research that identifies stigma as a primary barrier to formal help-seeking among KAs (Cheon et al., 2016). The finding that leaders in traditional, Korean-speaking ministries were least likely to make referrals further reinforces this interpretation, pointing to a potential ethnic insularity that can inhibit congregants’ bridging to external care, a phenomenon noted in studies on the social dynamics of immigrant churches (Park, 2013).

### Strengths, Limitations, and Future Directions

This study has several notable strengths. Its focus on KA church leaders addresses a significant gap in the literature on mental health in minority communities. Our framework drew on established models of mental health literacy (Jorm, 2012; Kutcher et al., 2016) and community gatekeeping (Isaac et al., 2009). By examining readiness, perception, and action as distinct dimensions, we identified unique predictors for each aspect of gatekeeping. This approach provided practical insights into which factors matter most for different types of mental health involvement among church leaders. Further, because the data were collected from KA church leaders across multiple regions, denominations, roles, and ministry contexts, this study provides practice-relevant insights into how mental health needs are identified, discussed, and managed within KA congregations. This study offers a detailed view into mental health concerns, stigma, and referral behavior in KA immigrant religious settings.

However, the study has limitations. First, the cross-sectional design prevents us from making causal claims; for example, we cannot definitively state that being second-generation causes a higher referral rate, only that there is a strong association. Second, while our sample of 93 leaders is robust, it was not randomly selected and may not be generalizable to all KA churches across different regions or denominations. Third, the data rely on self-report measures, which may be subject to social desirability bias (Fisher & Katz, 2000). Further, our survey did not directly assess barriers to mental health gatekeeping, and prior research indicates that ethnic and racial minority churches may also present challenges including hierarchical structures, confidentiality concerns, mental health stigma, and social pressure to save face (Avent et al., 2015; Baek et al., 2024). Future research should systematically examine both facilitators and barriers within Korean American church contexts to develop more effective and contextually appropriate interventions. Understanding how structural and cultural factors within churches may simultaneously enable and constrain mental health promotion efforts will be essential for developing sustainable partnerships.

These limitations point toward directions for future research. Longitudinal studies are necessary to track the impact of mental health training on leaders’ perceptions and actions over time. Future research employing longitudinal designs could also examine temporal relationships among these dimensions and test whether interventions targeting one dimension lead to changes in others. Qualitative research, such as in-depth interviews, would be invaluable for unpacking the *why* behind our central finding. Exploring the narratives and decision-making processes of bicultural leaders would provide a rich context for the quantitative data. Ultimately, comparative studies examining other Asian American or immigrant faith communities (Min & Jang, 2015) could help determine whether the observed patterns are unique to the KA church context or reflect broader dynamics within immigrant faith institutions.

### Implications

Despite its limitations, this study offers clear and actionable implications for churches, seminaries, and mental health practitioners. For faith communities and theological institutions, the findings highlight a need to integrate practical, skills-based mental health training into the core curriculum for clergy development. Given its impact on leader readiness, such training should be considered an essential component of pastoral formation. Furthermore, churches should recognize and intentionally empower their bicultural and bilingual leaders, not merely as ministers to younger generations, but as vital cultural bridges for the well-being of the entire community. Their unique positionality is a critical asset in connecting congregants to appropriate care, and they should be equipped and placed in positions of influence where this perspective can shape the church’s overall approach to mental health.

For mental health professionals, these findings suggest that Korean American churches hold potential as partners in community-based mental health efforts. Clinicians must actively build collaborative relationships with church leaders, recognizing them as key gatekeepers to an often-underserved population. This involves developing training materials and resources that are not only translated but are theologically integrated and culturally adapted for the immigrant church context. The finding that bicultural leaders are highly likely to make referrals identifies them as strategic partners for outreach. Clinicians with an understanding of the community’s faith values will be best positioned to build the trust necessary to receive these referrals and provide care.

## Appendix A

This appendix presents the survey questions used in this study. Below are the 16 substantive items analyzed in this study.

Q3: Location

State, city (i.e., Dallas, TX)

거주하고 계신 주, 시 (예시: 달라스, 택사스)

[Open-ended text response]

Q4: Church Denomination

교단.

[Open-ended text response]

Examples: Presbyterian, Baptist, Methodist, Nondenominational

Q5: Congregation Size

How large is your congregation?

본인 교회의 성도 규모를 선택해 주세요.

Response options:

- Less than 50 members [교인 50명 이하]
- 50–100 members [교인 50–100 명]
- 100–200 members [교인 100–200 명]
- 200–500 members [교인 200–500 명]
- Larger than 500 members [교인 500 명 이상]

Q6: Ministry Involvement

Which ministries do you have in your church? Check all that apply. Do you provide any special ministries?

교회 내 어느 사역을 섬기고 계신가요? 해당하는 선택지에 모두 선택해주세요. 또한 그 밖의 사역도 기타 란에 모두 적어주시길 바랍니다.

Response options (select all that apply):

- Korean Ministry [한국어 사역]
- English Ministry [영어 사역]
- Children’s Ministry [어린이 사역]
- Youth Ministry [청소년 사역]
- Missions Ministry [선교 사역]
- Other [기타]: _______________

Q7: Leadership Position

What church leader positions do you have in your church? Select all that apply.

교회 내 해당하시는 직책에 모두 선택 바랍니다.

Response options (select all that apply):

- Pastors [목사]
- Assistant pastors [부목사]
- Youth pastor [전도사]
- Elders [장로]
- Deacons [집사]
- Small-group leaders [소그룹 리더 및 목장장]
- Sunday school teachers [주일학교 교사]
- Other [기타]: _______________

Q8: Mental Health Training

Have you received some sort of mental health training in your seminary or elsewhere? If yes, where and for how long (months)?

이전 정신 건강 관련 교육 과정이나 훈련 과정을 밟으신 적이 있으신가요? 있으시다면 수료 기관명과 기간을 작성해주세요 (몇 월/ 몇 일).

[Open-ended text response]

Q9: Comfort Level (Readiness)

How comfortable do you feel addressing mental health issues in your church?

교회 내에서 정신 건강에 대한 대화를 다루는 것이 얼마나 편안하게 느껴지시나요?

Response options:

- Uncomfortable [불편함]
- Neutral [중도]
- Comfortable [편안함]
- Very comfortable [매우 편안함]

Q10: Settings for Mental Health Discussion

In what setting have you talked about mental health in your church? Select all that apply.

교회 내 정신 건강에 관한 이야기를 나눠보셨나요? 나눠주신 상황에 부합하는 선택지를 모두 선택 바랍니다.

Response options (select all that apply):

- Sermons [설교를 통해 나눔]
- Leaders or executive board [교회 지도자 및 행정부와 나눔]
- Leadership training [지도자 훈련 과정을 통해 나눔]
- Small group [소그룹 및 목장 모임 내에서 나눔]
- Individual [개별 대화에서 나눔]
- Other [기타]: _______________

Q11: Issues of Concern (Perception)

List ALL the issues that you are concerned about in your church.

교회와 교인들 가운데에 고민이 되는 이슈를 나눠주세요. 부합하는 선택지에 모두 선택 바랍니다.

Response options (select all that apply):

- Racism [인종차별]
- Domestic violence [가정 폭력]
- Marriage conflict [부부 갈등]
- Pressure to succeed [성공에 대한 압박/결과주의]
- Social media or screen addiction [디지털 미디어/SNS 중독]
- Depression and suicidal ideation [우울증과 자살]
- Anger management [분노 조절]
- Substance abuse [약물 남용]
- Alcohol misuse [알코올 오용]
- Sexual addiction including porn addiction [성 및 음란물 중독]
- Sexual abuse [성적 학대]
- Spiritual abuse [영적 학대]
- Mental health stigma [정신 건강에 대한 편견과 오해]
- Finance (lacking financial resource for counseling) [상담과 치료를 위한 금전적 문제]
- Burnout [번아웃 증후군]
- Intergenerational gap [세대 간 격차]
- Younger generations leaving the church [젊은 세대가 교회를 떠남]
- Problems with leadership transition [리더십 변화로 인한 교회 문제]
- Church split [교회 분열]
- Other [기타]: _______________

Q12: Barriers/Stumbling Blocks (Perception)

Select ALL the stumbling blocks you experience when handling the issues you selected on Q11.

질문지 11번에 대한 질문입니다. 왜 어렵게 느껴지시는지 해당 선택지에 모두 선택해주세요. 그 밖의 생각도 기타 란에 작성해주세요.

Response options (select all that apply):

- I don’t have enough time. [시간이 부족해서 어렵습니다.]
- I don’t know how to handle the issues. [어떻게 해결해야할 지 몰라서 어렵습니다.]
- It’s not my responsibility. [제가 해결할 의무를 가지고 있지 않습니다.]
- It takes too long to see a change. [변화를 위해서는 너무나 많은 시간이 필요합니다.]
- I would like to help out but I don’t have the authority or power. [(교인에게)도움을 제공하고 싶지만 제 교회 내 직책상의 한계가 있습니다.]
- I would like to help out but people don’t like to share about their problems. [(교인에게) 도움을 제공하고 싶지만 그 분들이 겪고 있는 문제를 털어놓지 못해 알 수 없었습니다.]
- We want to receive professional support, but we don’t have the financial means to do that. [저희 교회는 전문가 상담 및 지원 서비스를 제공하고 싶으나, 금전적 여유가 없습니다.]
- The issue can be only resolved through systematic changes. [해당 문제는 개인이 아닌 사회적·구조적 변화가 있어야만이 해결됩니다.]
- Other [기타]: _______________

Q13: Referral Behavior (Action)

Have you recommended professional counseling to anyone before?

주변 분에게 (교인이 아닌 분들도 포함) 정신 건강 상담을 추천한 경험이 있으신가요?

Response options:

- Yes [추천한 적이 있습니다.]
- No [추천한 적이 없습니다.]

Q14: Personal Counseling Experience

Have you received counseling before? If not, why?

정신 건강 상담을 받아보신 경험이 있으신가요? 없으시다면 이유를 나눠주세요. 기타 의견도 나눠주세요.

Response options (select all that apply):

- Yes, I have received counseling before. [네, 받아본 경험이 있습니다.]
- Counseling seems expensive. [상담 가격이 부담스럽거나 비싸서 받지 않았습니다.]
- I don’t know of any counselors. [상담사를 알지 못해 받지 않았습니다.]
- Other [기타]: _______________

Q15: Immigration Generation

What generation are you?

본인이 해당하는 세대에 선택 바랍니다.

Response options:

- 1st generation (You were born in Korea or outside the USA and moved to the USA as an adult) [1 세대: 한국이나 미국 밖 해외에서 태어나 성인이 된 후 미국으로 이주]
- 1.5 generation (You were born in Korea or outside the USA and moved to the USA before you turned 18 years old) [1.5세대: 한국이나 미국 밖 해외에서 태어나 18세 이전에 미국으로 이주]
- 2nd generation (You were born and raised in the USA) [2세대: 미국에서 태어나고 자람]
- 3rd generation (Your parents were born and raised in the USA and you were born and raised in the USA) [3세대: 부모님과 본인 모두 미국에서 태어나고 자람]

Q16: Preferred Language

What is your preferred language?

선호하는 언어를 선택해주세요.

Response options:

- Korean [한국어]
- English [영어]
- Fluent in both [두 언어 모두 유창]

Q17: Role Description

What is your role in the church?

교회 내 맡으신 역할이 무엇인가요?

[Open-ended text response]

Q18: Length of Service

How many years have you served in that role?

해당 역할을 섬겨주신 지 얼마나 되셨나요?

[Open-ended text response]

Q19: Additional Comments

Thank you so much for your time and invaluable answers. Please feel free to share any thoughts or questions to MSG.

귀한 시간 내어주셔서 감사합니다. 그 밖의 생각과 질문이 있다면 자유롭게 나눠주세요.

[Open-ended text response]

**Note:** Questions 9, 11, 12, and 13 were the primary variables analyzed in this study, measuring readiness (comfort level), perception (issues and barriers), and action (referral behavior), respectively.

## Footnotes

1 Missions Ministry was defined as outreach or evangelism efforts directed toward populations outside the Korean congregation, including both overseas and local mission activities (e.g., short-term mission trips, community service for non-Korean populations, or support for missionaries).

## References

[CR1] Abe-Kim, J., Takeuchi, D. T., Hong, S., Zane, N., Sue, S., Spencer, M. S., Appel, H., Nicdao, E., & Alegría, M. (2007). Use of mental health-related services among immigrant and US-born Asian Americans: Results from the National Latino and Asian American Study. *American Journal of Public Health,**97*(1), 91–98. 10.2105/AJPH.2006.098541 17138905 10.2105/AJPH.2006.098541 PMC1716256

[CR3] Avent, J. R., Cashwell, C. S., & Brown-Jeffy, S. (2015). African American pastors on mental health, coping, and help seeking. *Counseling and Values,**60*(1), 32–47. 10.1002/j.2161-007x.2015.00059.x

[CR4] Baek, K., Bell, C., Montgomery, S. B., Ortiz, L., Kumar, A., & Alemi, Q. (2024). Community-based mental health challenges and implications: Examining factors influencing distress and help-seeking behaviors among Korean American church leaders and members in greater Los Angeles. *International Journal of Environmental Research and Public Health,**21*(8), Article 1094. 10.3390/ijerph21081094 39200703 10.3390/ijerph21081094 PMC11354220

[CR5] Burnette, C., Ramchand, R., & Ayer, L. (2015). Gatekeeper training for suicide prevention: A theoretical model and review of the empirical literature. *Rand Health Quarterly,**5*(1), Article 16. 28083369 10.7249/rhq-05-01-16 PMC5158249

[CR6] Cheon, H.-S., Chang, E., Kim, P. Y., & Hyun, J. H. (2016). Mental health disparities impacting Christian Korean Americans: A qualitative examination of pastors’ perspectives. *Mental Health, Religion and Culture,**19*(6), 538–552. 10.1080/13674676.2016.1213712

[CR7] Fisher, R. J., & Katz, J. E. (2000). Social-desirability bias and the validity of self-reported values. *Psychology and Marketing*. 10.1002/(SICI)1520-6793(200002)17:2<105::AID-MAR3>3.0.CO;2-9

[CR8] Grenz, S. J. (2002). *The Baptist congregation: A guide to Baptist belief and practice*. Regent College Publishing.

[CR9] Han, S., Lee, H. S., & Kataoka, S. (2023). it’s taboo to talk about it”: Korean American clergy members’ views of mental health. *Psychiatric Services,**74*(10), 1096–1099. 10.1176/appi.ps.20220252 37042111 10.1176/appi.ps.20220252

[CR10] Hodge, A. S., Hook, J. N., Davis, D. E., & McMinn, M. R. (2020). Attitudes of religious leaders toward integrating psychology and church ministry. *Spirituality in Clinical Practice,**7*(1), 18–33. 10.1037/scp0000200

[CR11] Isaac, M., Elias, B., Katz, L. Y., Belik, S.-L., Deane, F. P., Enns, M. W., Sareen, J., & Swampy Cree Suicide Prevention Team. (2009). Gatekeeper training as a preventative intervention for suicide: a systematic review. *Canadian Journal of Psychiatry. Revue Canadienne de Psychiatrie*, *54*(4), 260–268. 10.1177/070674370905400407 10.1177/070674370905400407 19321032

[CR12] Jang, Y., Park, N. S., Yoon, H., Ko, J. E., Jung, H., & Chiriboga, D. A. (2017). Mental health literacy in religious leaders: A qualitative study of Korean American clergy. *Health and Social Care in the Community,**25*(2), 385–393. 10.1111/hsc.12316 26743312 10.1111/hsc.12316

[CR13] Jo, A. M., Maxwell, A. E., Yang, B., & Bastani, R. (2010). Conducting health research in Korean American churches: Perspectives from church leaders. *Journal of Community Health,**35*(2), 156–164. 10.1007/s10900-009-9213-1 20013060 10.1007/s10900-009-9213-1 PMC2835721

[CR14] Jorm, A. F. (2012). Mental health literacy: Empowering the community to take action for better mental health. *American Psychologist,**67*(3), 231–243. 10.1037/a0025957 22040221 10.1037/a0025957

[CR15] Jorm, A. F., Kitchener, B. A., Sawyer, M. G., Scales, H., & Cvetkovski, S. (2010). Mental health first aid training for high school teachers: A cluster randomized trial. *BMC Psychiatry,**10*(1), Article 51. 10.1186/1471-244X-10-51 20576158 10.1186/1471-244X-10-51 PMC2908569

[CR16] Kearns, K. P. (2006). Faith-based and secular social service agencies in Pittsburgh. *Journal of Community Practice,**14*(4), 51–69. 10.1300/J125v14n04_04

[CR17] Kim, S., & Kim, D. (2025). Mental health help-seeking among Korean men: The influence of stigma, masculine norms, and face. *BMC Psychology,**13*(1), Article 461. 10.1186/s40359-025-02793-y 40317029 10.1186/s40359-025-02793-y PMC12046740

[CR18] Kitchener, B. A., & Jorm, A. F. (2006). Mental health first aid training: Review of evaluation studies. *Australian and New Zealand Journal of Psychiatry,**40*(1), 6–8. 10.1080/j.1440-1614.2006.01735.x 16403032 10.1080/j.1440-1614.2006.01735.x

[CR19] Koh, E., Choi, G.-Y., Choi, S., & Cho, J.-Y. (2021). Korean immigrants’ perception of mental well-being and help-seeking behaviors. *Health and Social Work,**46*(3), 199–209. 10.1093/hsw/hlab009 34050666 10.1093/hsw/hlab009

[CR20] Kutcher, S., Wei, Y., & Coniglio, C. (2016). Mental health literacy: Past, present, and future. *Canadian Journal of Psychiatry. Revue Canadienne De Psychiatrie,**61*(3), 154–158. 10.1177/0706743715616609 27254090 10.1177/0706743715616609 PMC4813415

[CR21] Landes, S. D. (2010). Practicing discernment: Pastoral care in crisis situations. *The Journal of Pastoral Care and Counseling: JPCC,**64*(1), 4.1-8. 10.1177/154230501006400104 20827893 10.1177/154230501006400104

[CR22] Layzell, R. (2019). Pastoral response to congregational tragedy. In *Tragedies and Christian Congregations* (pp. 197–210). Routledge. 10.4324/9781351050791-19

[CR23] Lee, H. B., Hanner, J. A., Cho, S.-J., Han, H.-R., & Kim, M. T. (2008). Improving access to mental health services for Korean American immigrants: Moving toward a community partnership between religious and mental health services. *Psychiatry Investigation,**5*(1), 14–20. 10.4306/pi.2008.5.1.14 20046403 10.4306/pi.2008.5.1.14 PMC2796091

[CR24] Lee, J., Wachholtz, A., & Choi, K.-H. (2014). A review of the Korean cultural syndrome Hwa-Byung: Suggestions for theory and intervention. *Journal of Asia Pacific Counseling,**4*(1), Article 49. 10.18401/2014.4.1.4 25408922 10.18401/2014.4.1.4 PMC4232959

[CR25] Le Meyer, O., Zane, N., Cho, Y. I., & Takeuchi, D. T. (2009). Use of specialty mental health services by Asian Americans with psychiatric disorders. *Journal of Consulting and Clinical Psychology,**77*(5), 1000–1005. 10.1037/a0017065 19803580 10.1037/a0017065 PMC3938184

[CR26] Min, P. G., & Jang, S. H. (2015). The diversity of Asian immigrants’ participation in religious institutions in the United States. *Sociology of Religion,**76*(3), 253–274. 10.1093/socrel/srv025

[CR27] Liu, J. (2012, July 19). *Chapter 1: Religious affiliation*. Pew Research Center. https://www.pewresearch.org/religion/2012/07/19/asian-americans-a-mosaic-of-faiths-religious-affiliation/

[CR28] Matthieu, M. M., Cross, W., Batres, A. R., Flora, C. M., & Knox, K. L. (2008). Evaluation of gatekeeper training for suicide prevention in veterans. *Archives of Suicide Research,**12*(2), 148–154. 10.1080/13811110701857491 18340597 10.1080/13811110701857491

[CR29] Morgan, A. J., Ross, A., & Reavley, N. J. (2018). Systematic review and meta-analysis of Mental Health First Aid training: Effects on knowledge, stigma, and helping behaviour. *PLoS ONE,**13*(5), Article e0197102. 10.1371/journal.pone.0197102 29851974 10.1371/journal.pone.0197102 PMC5979014

[CR30] Park, J. Z. (2013). Ethnic insularity among 1.5- and second-generation Korean American Christians. *Development and Society*. 10.2139/ssrn.2736054

[CR31] Park, S. Y., Cho, S., Park, Y., Bernstein, K. S., & Shin, J. K. (2013). Factors associated with mental health service utilization among Korean American immigrants. *Community Mental Health Journal,**49*(6), 765–773. 23417654 10.1007/s10597-013-9604-8 PMC3976602

[CR32] Payne, J. S. (2014). The influence of secular and theological education on pastors’ depression intervention decisions. *Journal of Religion and Health,**53*(5), 1398–1413. 10.1007/s10943-013-9756-4 23846451 10.1007/s10943-013-9756-4 PMC4138430

[CR33] Pickard, J. G. (2012). Clergy perceptions of their preparation for counseling older adults. *Journal of Religion, Spirituality and Aging,**24*(4), 276–288. 10.1080/15528030.2012.683754

[CR34] Rumbaut, R. G. (2004). Ages, life stages, and generational cohorts: Decomposing the immigrant first and second generations in the United States. *International Migration Review,**38*(3), 1160–1205. 10.1111/j.1747-7379.2004.tb00232.x

[CR35] Stanford, M., & Philpott, D. (2011). Baptist senior pastors’ knowledge and perceptions of mental illness. *Mental Health, Religion & Culture,**14*(3), 281–290. 10.1080/13674670903511135

[CR36] United Methodist Church. (2016). *The book of discipline of the united Methodist church 2016*. United Methodist Publishing House.

[CR37] Vermaas, J. D., Green, J., Haley, M., & Haddock, L. (2017). Predicting the mental health literacy *of clergy: An informational resource for counselors*. *Journal of Mental Health Counseling,**39*(3), 225–241. 10.17744/mehc.39.3.04

[CR38] Wu, M. C., Kviz, F. J., & Miller, A. M. (2009). Identifying individual and contextual barriers to seeking mental health services among Korean American immigrant women. *Issues in Mental Health Nursing,**30*(2), 78–85. 10.1080/01612840802595204 19212865 10.1080/01612840802595204

[CR39] Wyman, P. A., Brown, C. H., Inman, J., Cross, W., Schmeelk-Cone, K., Guo, J., & Pena, J. B. (2008). Randomized trial of a gatekeeper program for suicide prevention: 1-year impact on secondary school staff. *Journal of Consulting and Clinical Psychology,**76*(1), 104–115. 10.1037/0022-006X.76.1.104 18229988 10.1037/0022-006X.76.1.104 PMC2771576

[CR40] Yamada, A.-M., Lee, K. K., & Kim, M. A. (2012). Community mental health allies: Referral behavior among Asian American immigrant Christian clergy. *Community Mental Health Journal,**48*(1), 107–113. 10.1007/s10597-011-9386-9 21249519 10.1007/s10597-011-9386-9

